# Knowledge and Attitude towards Obstructive Sleep Apnea among Primary Care Physicians in Northern Regions of Saudi Arabia: A Multicenter Study

**DOI:** 10.3390/healthcare10122369

**Published:** 2022-11-25

**Authors:** Abdullah N. Al-Rasheedi, Ashokkumar Thirunavukkarasu, Abdulhakeem Almutairi, Sultan Alruwaili, Hatem Alotaibi, Wasan Alzaid, Faisal Albalawi, Osama Alwadani, Ahmed Dilli

**Affiliations:** 1 Department of Otolaryngology and Head and Neck Surgery, College of Medicine, Jouf University, Sakaka 72388, Saudi Arabia; 2 Department of Community and Family Medicine, College of Medicine, Jouf University, Sakaka 72388, Saudi Arabia; 3 Department of Otolaryngology, Head and Neck Surgery, College of Medicine, Qassim University, Qassim 52571, Saudi Arabia; 4 College of Medicine, Jouf University, Sakaka 72388, Saudi Arabia

**Keywords:** primary care physicians, Saudi, knowledge, attitude, obstructive sleep apnea, referral

## Abstract

Obstructive sleep apnea (OSA) is a serious and often underreported condition, despite its highly prevalent distribution. Primary care physicians (PCPs) play an integral role in screening and managing patients with a high risk of developing OSA. This northern Saudi Arabian cross-sectional survey assessed the knowledge and attitude towards OSA among 264 randomly selected PCPs using the OSA Knowledge and Attitude (OSAKA) questionnaire. Among the participating PCPs, 43.9% and 45.1% had low scores in the knowledge and attitude categories, respectively. More than three-fourths (78%) of them recognized that an overnight sleep study is the gold standard for diagnosing OSA. Regarding referral, 39.4% of the OSA patients encountered by the PCPs were referred to ENT specialists, while 21% were referred to sleep clinics, and 18.2% were referred to pulmonologists. Nearly half (50.8%) of the participants recognized OSA as an important clinical disease, and 56.8% were confident in caring for OSA patients. Spearman’s correlation of the current study identified a positive correlation between knowledge scores and attitude scores (rho—0.151, *p* = 0.017). It is important to improve PCPs’ knowledge regarding OSA and the necessity for referral through different training methods. Furthermore, the study findings emphasize the need to include appropriate OSA programs and continuing medical education for PCPs.

## 1. Introduction

Sleep disorders are major public health issues, and their prevalence is increasing both in Saudi Arabia and globally [1,2]. Previous studies from Saudi Arabia observed a high prevalence of obstructive sleep apnea (OSA) among the general Saudi population, around 9%, and much higher among the special segment population, such as pregnant women. In general, the prevalence of OSA was higher in males than females [3,4,5,6]. This high prevalence of OSA correlates with the increasing obesity prevalence of about 35.6% among Saudi Arabians [3].

Sleep disruption is correlated with various daytime symptoms, such as daytime sleepiness, fatigue, and poor concentration. Furthermore, sufferers of OSA face higher risks of obesity and diabetes, as well as serious complications such as cardiovascular and cerebrovascular events [7,8]. A recent analysis estimated that 936 million individuals of both genders aged 30–69 years were found to have obstructive sleep apnea worldwide [9]. In Saudi Arabia, clinically diagnosed sleep apnea affects 8.5% of the population [3].

OSA is a serious and often underreported condition, despite its highly prevalent distribution. OSA’s rising prevalence is progressively emerging as a global health epidemic, primarily as a result of the prevailing obesity epidemic [10]. A recent systematic review of 24 studies reported a wide range of OSA prevalence (apnea–hypopnea index, AHI ≥ 5) ranging from 9 to 38% [11]. Moreover, Chung et al. suggested that primary care physicians (PCPs) had insufficient knowledge of OSA, resulting in the underestimation and underdiagnosis of OSA [12].

PCPs are integral in screening and managing patients with several diseases, including OSA [13]. Patients with OSA symptoms often present in primary health care settings such as primary health centers (PHC) [14,15]. As such, many studies have been performed to assess PCPs’ knowledge, attitude, and practice (KAP) regarding OSA, which showed variable results ranging from good to poor knowledge and attitudes. Furthermore, the ability to manage OSA among PCPs, even in those with good knowledge, was inadequate [14,16].

Several studies have been conducted to evaluate physicians’ knowledge of OSA in several respects. Knowledge scores varied according to different specialties, where the highest score of 88.9% was seen among otolaryngology-head and neck surgery residents [17] and the lowest score of 64% was seen in PCPs [14]. Furthermore, Al-Khafaji et al., 2021, reported that higher knowledge scores were accomplished by physicians who have access to sleep centers (13.2 SD 2.2, *p* < 0.001) [16]. In a previous study in Saudi Arabia, Al-Saleem et al., 2020, estimated PCPs’ knowledge level of OSA from the Al-Hasa region, which was about 55% [18].

The steps used to reach the diagnosis and initiation of treatment of OSA usually start in a PHC setting, followed by referral to secondary care, as OSA usually requires specialist input [19]. Devani et al., 2020, introduced a model for collaboration across primary and secondary care for patients with suspected OSA. This pathway allows patients to undergo a preliminary assessment and receive a sleep study device at a PHC setting while maintaining specialist input through a virtual multidisciplinary team [19].

Many studies have emphasized the importance of assessing PCPs’ knowledge regarding OSA [15,16]. Therefore, investigating the gaps in this knowledge is important in the design of educational strategies. There is a wide variation in sociocultural and health-related characteristics among the Saudi population, as well as in different regions. Furthermore, most training programs are conducted in major cities. Hence, PCPs in the northern region might not have sufficient opportunities to attend the required training programs related to obesity and OSA. There has been an alarming increase in the prevalence of obesity in the Middle East, and OSA is one of the major complications that needs timely attention from PCPs. From our extensive literature search in major databases, the authors could not find sufficient data in this context in the northern regions of Saudi Arabia. Considering the need for region-specific data for formulating a necessary policy for the required training program related to OSA, the present study was conducted. The current study explores the knowledge and attitude towards OSA among PCPs in the northern regions of Saudi Arabia.

## 2. Materials and Methods

### 2.1. Study Description

The present survey is an analytical, cross-sectional study that was carried out in PHC settings in the Aljouf, Northern Borders, and Tabuk regions of Saudi Arabia. PCPs (i.e., general practitioners, family doctors, and family residents) were invited to participate in the study. This study was carried out from 1 February 2022 to 30 August 2022.

### 2.2. Inclusion and Exclusion Criteria

All PCPs working at PHCs in these regions and willing to participate in the study were included in the study. The PCPs who were on vacation and unwilling to participate were excluded from the study.

### 2.3. Sample Size

**Figure 1:**
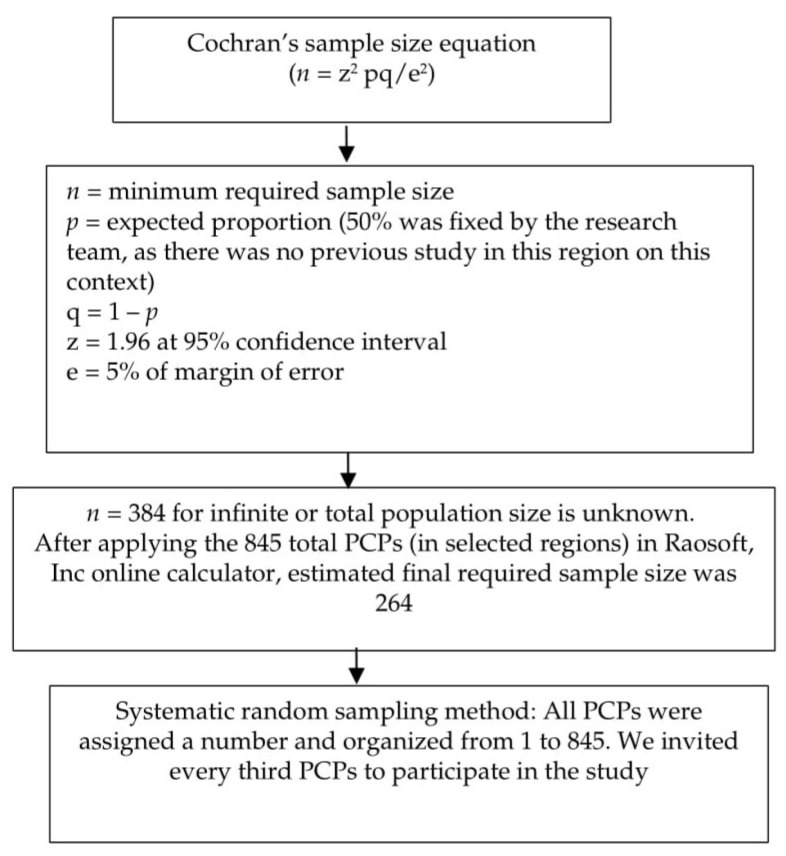
Sampling strategy flowchart.

The present study utilized Cochran’s sample size equation (*n* = z^2^ pq/e^2^), a widely used sample size estimation formula [20]. The sample size was calculated based on the level of sufficient knowledge of OSA among PCPs, representing 50% with a 5% margin of error and 95% confidence interval, as well as 80% power for the study. The estimated sample size was 384 participants. However, after applying it to the total number of PCPs in these three regions (a finite population), the final estimated minimum required sample size was 264. A systematic random sampling strategy was incorporated to select the necessary participants. The sampling strategy utilized in the present cross-sectional study is described in the flow chart below (Figure 1).

### 2.4. Data Collection

We collected data after obtaining an ethical approval letter from a regional health affairs ethics committee (Qurrayat, Wide approval no. 120). All the PCPs agreed to participate in the present study through informed consent. This study was conducted using a self-administered questionnaire comprising two parts.

#### 2.4.1. Part I: Personal Information

This part of the questionnaire contains sociodemographic data, including age, gender, nationality, highest qualification, current position, years in practice, number of OSA cases encountered during the past year, and the referring department.

#### 2.4.2. Part II: The Obstructive Sleep Apnea Knowledge and Attitude (OSAKA) Questionnaire

We used the English version of the OSAKA questionnaire to assess PCPs’ knowledge and attitudes towards OSA. This data-collection instrument was initially constructed and validated in the United States to assess physicians’ knowledge and attitudes regarding the identification and management of patients with OSA [21]. The OSAKA questionnaire is a self-administered survey that takes only a few minutes to complete. It comprises the following parts:

*The first part*: This contains eighteen questions that assess five domains (epidemiology, pathophysiology, symptoms, diagnosis, and treatments). Knowledge items are presented as yes, no, and do not know options. The score for the correct answer was given as one; the other choices were marked as zero.

*The second part*: This constituted five items. The first is developed to measure the importance of OSA as an important clinical disease. The second item assessed the importance of identifying OSA patients by their PCPs. The remaining three items measured the self-confidence of PCPs in the management of OSA patients. The participants responded to these three items on a 5-point Likert scale, ranging from 1 to 5 (1—strongly disagree to 5—strongly agree). Both knowledge and attitude scores were further classified into low (<60% of the overall score), average (60 to 79% of the overall score), and high (≥80% of the overall score). This category was included according to Bloom’s criteria and has been used by several studies in the past [22,23].

### 2.5. Statistical Analysis

We used the statistical package for social sciences (SPSS), V.21 (IBM, SPSS Inc., Chicago, IL, USA) for data entry, coding, and analysis. The present study used frequency with proportion to show categorical data and mean and median to depict quantitative data. The Shapiro–Wilk test was used to test the data’s normality assumption. We applied Spearman’s analysis to find the correlation between knowledge and attitude scores. Furthermore, we executed a Chi-square test for categorical background characteristics of the PCPs and a Wilcoxon rank sum test for age and practice duration of the PCPs to find the association between the knowledge categories. We have set the *p*-value of less than 0.05 as a significant value. Finally, all of the statistical tests that were run were two-tailed.

## 3. Results

The total number of PCPs who participated in this study was 264, and these participants were selected using a systematic sampling strategy. The present survey’s participants’ demographic characteristics and education details are described in Table 1. Among the PCPs who participated, 48.1% were males and 51.9% were females, with a mean (SD) age of 33 (8.01). Nearly half (47.7%) of them had only undergraduate (MBBS) qualifications, and 57.9% of them were residents with a mean (SD) practice duration of 8 (6.6) years.

Regarding referral patterns, 104 (39.4%) OSA patients encountered by the physicians were referred to ENT specialists, 21.0% were referred to a sleep clinic, and 18.2% of patients were referred to pulmonologists (Table 2).

**Figure 2:**
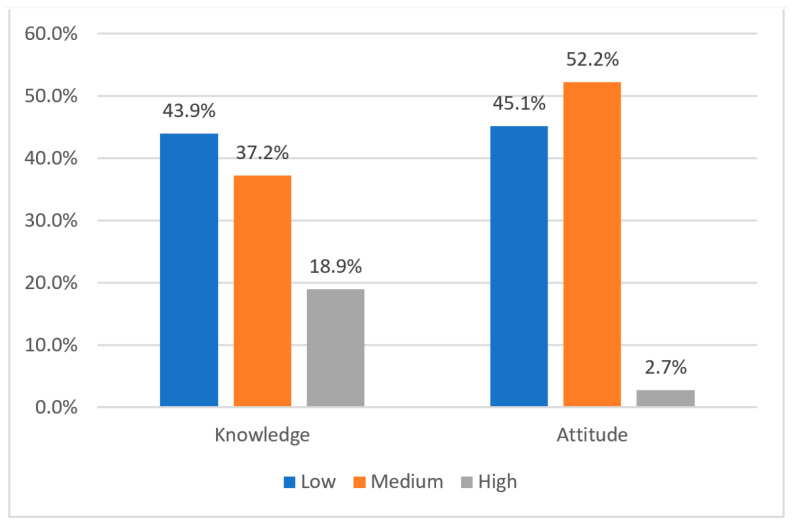
Knowledge and Attitude Categories (*n* = 264).

Figure 2 shows the categories of knowledge and attitude scores, as per Bloom’s category. Among the sampled physicians, 43.9% had a low score in knowledge, while 18.9% had high scores. Regarding attitude, 45.1% had a low score and only 2.7% had a high score.

Table 3 depicts the proportion of correct answers given by the studied PCPs. The current survey found that the highest proportion of the correct answer was found for the statement “In children, adenoids and large tonsils most commonly cause OSA (81.8%)”, followed by the statement “Snoring is present in most of the patients with OSA (81.4%)”, and “An overnight sleep study is a gold standard for diagnosing OSA (78.0%)”.

When assessing the association between PCPs’ sociodemographic and background characteristics, we found a significant association among different regions (*p* = 0.002) in terms of knowledge categories. No other factors were significantly associated with the knowledge categories (Table 4).

Regarding the importance of the attitude section, nearly half (50.8%) of the participants recognized OSA as an important or very important clinical disease. Additionally, 51.5% of the PCPs considered identifying people with OSA to be very critical. Regarding self-confidence, 73.9% of the participants were confident in finding people with OSA, and 56.8% were confident in caring for OSA patients (Table 5).

Spearman’s correlation of the current study identified a positive (weak) correlation between knowledge scores and attitude scores (rho—0.151, *p* = 0.017) (Table 6).

## 4. Discussion

The proper management of sleep disorders, including OSA, could help prevent several chronic diseases. The role of PCPs is vital in diagnosing and referring patients to secondary care, as most cases require this step. Our survey assessed PCPs’ knowledge and attitudes towards OSA and its referral patterns.

Our study results explored a wide variation in the knowledge questionnaire items, ranging from 36.0% to 81.8%, which is comparable to the studies performed by Corso et al. and Embarak et al. [24,25]. This result demonstrates that knowledge regarding OSA is inadequate and needs appropriate curriculum training. Our study outlined that the highest proportion of correct answers from PCPs was for the item related to snoring. This finding is in alignment with an Egyptian study conducted in 2020. In this study, the majority (79.7%) of the participants also agreed that most patients with OSA were snorers. The present study reported that the proportion of median (IQR) total knowledge scores of PCPs was 59.4 (33.3%). Recent studies conducted in Ecuador and Saudi Arabia also reported a similar finding, and their results found that the respondents’ mean score was around 10 out of 18 [18,26]. However, some other studies have reported slightly higher knowledge mean scores among participants [14,24]. The most likely factors for the dissimilarities in the results could be the study settings and the inclusion of participants. Regarding referral patterns, PCPs most commonly referred suspected and diagnosed OSA cases to an ENT specialist; Devaraj et al. also found similar results [19].

Knowledge regarding the association between hypertension and OSA is essential for physicians. The present study revealed that only two-thirds of the PCPs answered correctly to the statement “OSA is associated with hypertension”. Interestingly, a cross-sectional study conducted by Cherrez et al. found that a lower proportion (less than 50%) of the physicians responded with correct answers to the above statement [27]. The present study found that a lower proportion of the participants answered correctly to the statement, “Continuous positive airway pressure (CPAP) is the first line of therapy for severe OSA management”. Similar to our findings, numerous recent surveys have explored a lower level of knowledge regarding this statement among their study participants [27,28,29,30]. Our study findings and studies from other countries indicate that PCPs have insufficient knowledge regarding OSA, which could decrease their ability to diagnose and refer patients to a specialist for necessary care. Regarding the identification of associated factors for the PCPs’ knowledge of OSA, we did not find any significantly associated factors, except for regional distinctions.

Numerous existing texts suggest that self-confidence is an essential feature of a great physician, including PCPs [31,32,33]. This northern Saudi Arabian survey revealed that only a low proportion of the PCPs were confident in caring for OSA patients (56.8%), and the same was true regarding their ability to handle OSA patients on CPAP. Similar to this study’s findings, another study by Chang et al. compared PCPs’ attitudes toward OSA in three African regions, and they reported that their study participants had low confidence in caring for OSA patients [28]. However, an Italian study by Corso et al. noted that a higher proportion of their participants had confidence in the items mentioned. The possible variations in the study findings could be due to the differences between the respective study participants [24]. Both our study and the study by Chang et al. conducted the survey using the OSAKA questionnaire, whereas the latter survey was conducted among anesthesia specialists. Al-Khafaji et al. 2021, assessed the confidence of Middle Eastern and North African physicians of different specialties’ in managing patients with OSA; they found that 72% of practitioners in internal medicine and 49% of practitioners in family medicine/general medicine were confident in managing patients with OSA [16]. However, most primary care physicians executed referrals for patients with suspected OSA to otorhinolaryngologists (72–83%) [14,28]. In addition, only 10% of PCPs used screening tools for OSA, and only 16% of PCPs used the latest clinical practice guidelines, according to Devaraj et al. in a 2020 study [14].

A positive attitude among healthcare workers is critical for patient care. The present study explored a positive correlation between knowledge and attitude, assessed by Spearman’s correlation test (Spearman’s rho = 0.151, *p* = 0.014). Similar to the present study’s results, Al-Khafaji et al., in 2021, and Odeja et al., in 2019, also revealed the same findings [16,27].

## 5. Strengths and Weaknesses of the Present Study

The current survey is the first of its kind in the northern region of Saudi Arabia and was conducted recently. We also used a standard and validated data collection tool. However, cross-sectional and questionnaire-related survey biases, such as self-selection bias, could have influenced the results. Additionally, the study is limited to northern regions, and the findings may not be suitable for other regions of Saudi Arabia.

## 6. Conclusions

The present study explored inadequate knowledge of OSA among primary care physicians, and we also examined a positive correlation between knowledge and attitude among PCPs. Hence, improving PCPs’ knowledge regarding OSA and the necessity for referral in this region arise recommended through the use of different training methods. Furthermore, the study findings highlight the need to include appropriate OSA programs and CME for PCPs, effective OSA education for undergraduates, and enhanced residency training with the provision of sleep medicine rotations, lectures, and workshops on OSA screening, diagnosis, and management.

## Figures and Tables

**Table 1:** Demographics and participants’ characteristics (*n* = 264).

Characteristic	Frequency	Percentage
Gender		
Male	127	48.1
Female	137	51.9
Age in years, mean (SD)	33 (8.01)	
Regions		
Aljouf	59	22.3
Tabuk	89	33.7
Northern border	116	43.9
Latest qualification		
MBBS	126	47.7
MD	69	26.1
Board certified	42	15.9
Others	27	10.2
Current position		
Residents	153	57.9
Specialists/Registrars	63	23.9
Consultants	48	18.2
Practice duration in years, mean (SD)	8 (6.6)	
Rate OSA Cases encountered Mean (SD)	2.63 (2.22)	

**Table 2:** Referral patterns of primary care physicians (*n* = 264).

Specialty	Frequency	Percentage
ENT	104	39.4
Sleep clinic	57	21.0
Respiratory	48	18.2
Neurology	13	4.9
Pediatrics	4	1.5
Others	25	9.5
None	13	4.9

**Table 3:** Proportion of correct answers given by the study participants for each knowledge section item.

Question Number	OSAKA Questions	Correct Answer *n* (%)
1	Females with OSA (obstructive sleep apnea) may have only fatigue	149 (56.45)
2	Uvulopalatopharyngoplasty is a cure for most patients with OSA	130 (50.8)
3	Adult OSA prevalence is estimated to be between 2 and 10%	108 (40)
4	Snoring is present in most of the patients with OSA	215 (81.4)
5	OSA has an association with hypertension	175 (66.3)
6	The gold standard for the diagnosis of OSA is overnight sleep study	206 (78)
7	CPAP therapy may lead to nasal congestion	163 (61.7)
8	Laser-assisted uvuloplasty is an appropriate treatment for severe OSA	134 (50.8)
9	OSA may be due to a loss of upper airway muscle tone during sleep	184 (69.7)
10	In children, adenoids and large tonsils most commonly cause OSA	216 (81.8)
11	A useful examination in suspected OSA is a craniofacial and oropharyngeal examination	193 (73.1)
12	Alcohol at bedtime improves OSA	81 (30.7)
13	Untreated OSA has an association with a higher incidence of car related accidents	180 (68.2)
14	A collar size greater than 17 is associated with OSA in males	108 (40.9)
15	Females suffer from OSA more than males	141 (53.4)
16	CPAP is the first therapy for severe OSA	92 (34.8)
17	In adults, it is normal to have five apneas or hypopneas in one hour	95 (36)
18	There may be an association between untreated OSA and arrhythmias of the heart	171 (64.8)
	Median (IQR) of the correct answer (%) in the knowledge category	59.14 (33.3%)

**Table 4:** Relationship between knowledge and background characteristics of the PCPs (*n* = 264).

		Knowledge		
	Total (264)	Low/Average (214) *n* (%)	High (50) *n* (%)	*p*-Value
Gender *				
Male	127	107 (84.3)	20 (15.7)	0.213
Female	137	107 (78.1)	30 (21.9)	
Age in years: Mean (SD) **		33.40 (8.41)	32.98 (8.11)	0.744
Regions *				
Aljouf	59	51 (86.4)	8 (13.6)	0.002 ***
Tabuk	89	81 (91.0)	8 (9.0)	
Northern border	116	81 (70.7)	34 (29.3)	
Latest qualification*				
MBBS	126	97 (77.0)	29 (23.0)	0.056
MD/MS	69	58 (84.1)	11 (15.9)	
Saudi Board certified	42	33 (78.6)	9 (21.4)	
Others (Fellowship, PhD)	27	26(96.3)	1 (2.0)	
Current position *				
Residents	153	121 (79.1)	32 (20.9)	0.585
Specialists/Registrars	63	52 (82.5)	11 (17.5)	
Consultants	48	41 (85.4)	7 (14.6)	
Practice duration in years, mean (SD) **		7.99 (6.55)	6.82 (5.68)	0.267

* Chi-square test applied; ** independent t test applied; *** Significant association.

**Table 5:** PCPs’ responses and associated factors in the attitude section (*n* = 264).

Item	Frequency	Proportion
Importance of identifying OSA patients (data shown are either important or very importance)		
OSA is an important disease	134	50.8
Diagnosis of people with OSA is very much essential	136	51.5
Self-confidence (data shown are either agree or strongly agree)		
Confident in diagnosing patients with a high risk of developing OSA	195	73.9
Confident in their capability to care for OSA patients	150	56.8
Confident in their skills to manage OSA patients on CPAP treatment	125	47.3

**Table 6:** Correlation between knowledge and attitude scores, assessed by Spearman’s correlation test.

	Spearman’s Coefficient Value (Rho)	*p*-Value
Knowledge–Attitude	0.151	0.017 *

* Significant value.

## Data Availability

The raw SPSS data used in the present study is available upon request from the corresponding author.
